# Flap reconstruction in rectal resection and exenteration surgery: a single centre retrospective cohort study

**DOI:** 10.3389/fsurg.2026.1710035

**Published:** 2026-02-19

**Authors:** Jonathan Tebabu Wubetu, Valentin Butnari, Ahmer Mansuri, Gursharan Paul Singh Bawa, Baskaran Sabapathipillai, Richard Boulton, Saswata Banerjee, Matthew Hanson, Joseph Huang, David Burling, Sandeep Kaul, Manu Sood, Rishabh Bassi, Waseemullah Khan, Nirooshun Rajendran

**Affiliations:** 1 General Surgery Department, Queens Hospital, NHS Barking, Havering and Redbridge Trust, Essex, United Kingdom; 2 Centre for Neuroscience, Surgery & Trauma, Blizard Institute, Queen Mary University London, London, United Kingdom; 3 Intestinal Imaging Centre, St Mark’s Hospital, Harrow, United Kingdom; 4 Plastic and Reconstructive Surgery Department, Mid and South Essex NHS Foundation Trust, Essex, United Kingdom

**Keywords:** abdomino perineal excision (APE), abdomino perineal resection, ELAPE, extralevator abdominoeperineal excision (ELAPE), flap reconstruction, multidisciplinary care, rectal cancer

## Abstract

**Purpose:**

To evaluate the outcomes of flap reconstruction following extralevator abdominoperineal excision (ELAPE) compared to abdominoperineal resection (APR) in the treatment of locally advanced and recurrent rectal cancer, in the context of demonstrating the feasibility of performing ELAPE with flap reconstruction for rectal cancer in a large public (non-tertiary) hospital. The primary outcome was the assessment of postoperative complication rates to determine whether outcomes fell within acceptable standards for complex pelvic reconstruction. Secondary outcomes included flap-specific complications, operative parameters, postoperative length of stay, and correlations between flap complexity, operative duration, complication grade, and recovery metrics.

**Methods:**

This retrospective cohort study analysed 39 patients who underwent reconstructive ELAPE or APR at a secondary referral centre between April 2018 and August 2024. Data were collected from a prospectively maintained database and validated using clinical records and MDT meeting summaries. Patient demographics, surgical details, flap types, postoperative outcomes, and complication rates were evaluated. Statistical analyses included descriptive statistics and correlation assessments.

**Results:**

Twenty-seven patients underwent ELAPE with flap reconstruction, utilizing vertical rectus abdominis myocutaneous (VRAM), inferior gluteal artery (IGAM), gracilis, and V-Y advancement flaps. Outcomes, including complication rates and length of hospital stay, were comparable to those reported by tertiary centres. Major complications (Clavien-Dindo grade III and above) occurred in 33.3% of ELAPE cases, with flap-specific complications such as superficial infections (14.8%) and dehiscence requiring intervention (7.4%). Median length of stay for ELAPE was 18 days. No cases of complete flap failure were observed.

**Conclusion:**

This study demonstrates that ELAPE with flap reconstruction can be safely and effectively performed in a large public hospital setting, with outcomes comparable to high-volume tertiary centres. The findings underscore the importance of multidisciplinary collaboration in achieving high-quality surgical and reconstructive outcomes, and how these can be achieved in a large public hospital.

## 1 Introduction

Colorectal cancer (CRC) remains a significant contributor to global morbidity and mortality, causing an estimated 881,000 deaths worldwide, including approximately 331,000 attributed to rectal cancer alone (1). In the United Kingdom, rectal cancer presents a major health burden, with a notable percentage of patients presenting with low rectal tumours often requiring major surgical resection (2). Abdominoperineal excision (APR) has historically been the standard treatment, but concerns over high rates of circumferential resection margin (CRM) involvement have driven the development of more radical and patient tailored techniques (3).

The Extralevator Abdominoperineal Excision (ELAPE) represents a significant evolution in the surgical management of this condition. Introduced to address the limitations of conventional APR, ELAPE involves a wider excision that includes the levator muscles, aiming to produce a cylindrical specimen, reduce CRM positivity, and subsequently improve oncological outcomes (4, 5). The radical nature of ELAPE, and indeed some extensive APRs, often results in a large and complex perineal defect. Effective closure of this defect is critical to prevent significant complications such as wound dehiscence, chronic infection, and perineal herniation, mandating the involvement of plastic surgery for advanced reconstructive techniques (6, 7).

Planning these procedures and overall successful management of locally advanced and recurrent rectal cancer relies on a highly specialised multidisciplinary approach. Radiologists play a vital role in this process by providing a detailed so called ‘roadmap’ which represents a interpretation of preoperative MRI that guides the team in both the resectional and reconstructive components of surgery (7). One of the most valuable tools for comprehensive pre-operative assessment and subsequent planning of individualized reconstructive surgery that radiologists use represents concept-mnemonic ‘BONVUE’ (Bones, Organs, Nerves, Vessels, Ureters, and Extra-tumoral sites) (7).

This tailored resection often results with large perineal defect. Closure of the pelvic floor and perineum after these procedures involves a high risk of flap-specific morbidity; moreover, these risks are increased as most of these patients would have received preoperative radiotherapy. To achieve optimal outcomes, the management strategy must be a collaborative effort between colorectal surgery, plastic surgery, and a host of other specialties, including oncology and radiology. The choice of type of closure for perineal reconstruction after ELAPE or APR depends on the size of the defect, patient habitus, and the availability of donor tissue (8). The most common flap types employed include the robust Vertical Rectus Abdominis Myocutaneous (VRAM) flap (based on the deep inferior epigastric vessels) (9), the versatile gluteal flaps [such as the Inferior/Superior Gluteal Artery Perforator (IGAP/SGAP) flaps] (10, 11), and the less bulky Gracilis muscle flap (11, 12). While detailed descriptions of the surgical harvest of these flaps are numerous in the literature, their primary role is to obliterate the resulting dead space, provide vascularised tissue coverage, and reduce the risk of flap specific and overall postoperative complications.

This study aims to evaluate the outcomes of patients undergoing ELAPE and APR with concomitant flap reconstruction. Our centre, a large public hospital, collaborates closely with a tertiary institution in Essex for specialised plastic surgery and benefits from expert London-based radiological and multidisciplinary support. The primary objective is to showcase the reconstructive surgery outcomes post-ELAPE and compare them to classical APR surgery within this non-tertiary healthcare setting. The majority of published evidence regarding complex perineal reconstruction following radical pelvic surgery originates from highly specialised, high-volume tertiary centres (13, 14). The novelty and emphasis of this paper lie in demonstrating that excellent, comparable reconstructive outcomes can be achieved in a secondary, non-tertiary hospital setting through robust multidisciplinary collaboration and adherence to established surgical principles. By presenting our experience and focusing on the technical aspects, flap outcomes, and associated complications, we aim to contribute to the body of evidence supporting the feasibility and efficacy of advanced reconstruction for locally advanced rectal cancer in a broader range of clinical environments.

## 2 Materials and methods

### 2.1 Study design, patient selection and lexicon

- Data were obtained retrospectively between April 2018 and August 2024, through interrogation of a prospectively maintained electronic database, systematically created at a single secondary referral centre undertaking advanced pelvic and coloproctological oncological resection with perineal reconstruction. Data were collected for patients undergoing aforementioned exenterative and reconstructive APR and ELAPE surgery for locally advanced/recurrent rectal cancer. Procedures with non-curative intent were included if those patients underwent reconstructive APR/ELAPE. Patients who underwent APR without reconstruction were excluded. When required, the database data were corroborated by retrospective review of operative details, clinical data, electronic health records, pathology reports, electronic discharge summaries, clinical correspondence, notes from multidisciplinary team (MDT) meetings, clinic letters, radiological imaging, and inpatient medical notation. Desired data points included (but were not limited to) patient demographics; flap type; extent of resection/exenteration; local vs. recurrent disease; histology; initial and final TNM staging; use of neo-adjuvant/adjuvant chemoradiotherapy; resection margin status; laparoscopic/open approach; estimated blood loss; post-operative complications (via Clavien-Dindo classification); survival time; time of death.
- Definitions and classifications used within this study adhere to those defined by the Lexicon Collaboration of The United Kingdom Pelvic Exenteration Network (UKPEN) and the Association of Coloproctology of Great Britain and Ireland (ACPGBI) Advanced Cancer subcommittee (15).
- This study was conducted and reported in alignment with the ‘Preferred Reporting of Case Series in Surgery’ (PROCESS) guidelines, and was registered according to local governance protocols, ensuring that data collection adhered to data protection regulations. Caldicott guardianship within NHS BHRUT allowed ethical approval for this audit, noting that no patient-identifiable information was used in this study (16).

### 2.2 Staging and surgery

- This study observed a standardised approach to radiological staging and surgical planning for coloproctological malignancy. Utilisation of computed tomography (CT) and positronic emission tomography (PET) was uniform throughout the ELAPE/APR cohort, providing in-depth anatomical assessment and staging. Multitudes of studies and guidelines place pelvic magnetic resonance imaging (MRI) as the gold standard for mesorectal assessment with relevance to the total mesoractal excision (TME) plane, TNM staging, and assessment of extent of local pelvic infiltration (17). Total seven-compartment staging permits surgical planning relevant to the UKPEN and ASCGBI lexicon, allowing the accurate determination and classification of structures requiring resection. This discussion is considered by the complex colorectal MDT, in conjunction with patient-individualised discussions surrounding neo-adjuvant therapy in relation to demographics and malignancy characteristics. Observed neo-adjuvant therapies included long-course chemoradiotherapy (CRT) and short-course radiotherapy. Patients who have received neo-adjuvant treatment typically undergo restaging in a similar radiological fashion to initial staging, thereby providing information on tumour response. Re-discussion at MDT facilitates discussions around ongoing management, including surgical resection if required. Indications for exenterative and reconstructive APR and ELAPE surgery are as follows: 1. histologically proven and resectable primary adenocarcinoma of the low rectum initially staged T3–4, N0–2; 2. external sphincter muscle involvement; 3. MDT-Perceived impossibility to achieve a safe distal margin with a sphincter sparing technique (18, 19). However, not all patients were eligible for surgery. Patient preference was considered. Those not offered surgical intervention included poor functional status/those deemed surgically unfit, those with a lack of reconstructive options, or those possessing distantly disseminated metastatic disease.

### 2.3 Perioperative outcomes

- Perioperative outcomes, including transfusion needs and short-term surgical complications, were extracted from all correspondence within the 30-day postoperative period and classified (as previously mentioned) using the extended Clavien-Dindo classification system. These had been prospectively logged within the aforementioned database. The length of hospital stay, and 90-day mortality were also recorded.

### 2.4 Follow-up

- Follow-up duration was defined as the interval between the surgical procedure and the date of the most recent documented clinic visit, in patient discharge, or death.

### 2.5 Data analysis

- All statistical analyses were performed using GraphPad Prism [Version 10.3.0 (461), GraphPad Prism Software, U.S.A]. Analyses included descriptive statistics (continuous variable descriptors including mean, median and range; categorical variable descriptors such as percentage and frequency). Depending on data distribution, differences between groups were examined using a combination of parametric and non-parametric methods. Analysis of variance (ANOVA), including Welch and Brown–Forsythe corrections where appropriate, was used to compare continuous operative and postoperative outcomes across the six flap types. Paired t-tests were employed for within-patient comparisons where relevant paired measurements were available. Comparisons of categorical variables between groups were performed using Fisher's Exact test to account for the small sample size and the presence of low expected cell frequencies across complication categories. Spearman's rank correlation coefficient was used to assess monotonic associations between key perioperative variables, particularly where non-normality or ordinal data were present. For correlative analyses and ANOVA involving flap type, flaps were ranked according to objective operative complexity (1 = least complex, 6 = most complex) to facilitate analysis and interpretation of linear trends across the spectrum of reconstructive options. This allowed for improved interpretation of correlative multivariate tests i.e., understanding positive or negative correlative relationships with regards to flap type.
- This flap hierarchy, although novel and subjective, is consistent with published descriptions identifying gracilis and V-Y advancement flaps as relatively straightforward options, and larger myocutaneous flaps, such as IGAM and VRAM and combined or bilateral reconstructions, as more complex procedures typically used for extensive defects. This is discussed further in our discussions section below.

### 2.6 Primary and secondary outcomes

- Primary outcome: To evaluate whether postoperative complication rates following flap-reconstructed ELAPE in our large public hospital fall within the range of outcomes considered acceptable in the published literature, thereby assessing the feasibility and safety of delivering complex, tertiary-level rectal reconstructive cancer surgery in a non-tertiary setting. Contextual comparison with results reported by tertiary centres is undertaken in the Discussion to benchmark our findings against established standards.
- Secondary endpoints*:* Secondary outcomes included: (1) flap-specific complication profiles, such as superficial infection, dehiscence, necrosis, and the need for negative-pressure therapy or interventional radiology drainage; (2) operative metrics, including total surgical duration and estimated blood loss; (3) postoperative recovery metrics, prima§rily length of hospital stay; and (4) exploratory correlations between flap complexity, postoperative length of stay, Clavien–Dindo complication grade, and operative duration.

## 3 Results and discussion

### 3.1 Patient characteristics

**Figure 1:**
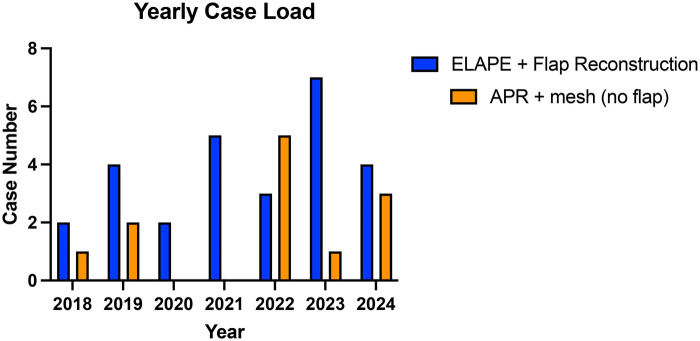
Yearly case numbers of both ELAPE and APR surgery within barking, havering and redbridge NHS trust (NHS BHRUT).

There were 39 patients, with a mean age of 56.7 years (range 34–80), and 20 (51.3%) were males. Case distribution for the study period is represented in Figure 1 incorporating the COVID-19 Pandemic. Study patient characteristics are summarised in Table 1. Study groups are uniform with no statistical differences found during analysis of cohort age, gender, BMI, Race, ASA, Approach, or neo-adjuvant therapy used (Table 1).

**Table 1:** Demographics of the ELAPE/APR cohorts.

Characteristic	Total (Range/percentage)	ELAPE (with flap)	APR (with mesh recon	Group difference (P)
No. of patients	39	27	12	–
Age (in years)	60.3 (34–80)	58.3 (33–76)	64.9 (44–80)	0.176
Sex				
Women	19 (48.7)	10 (37.0)	9 (75.0)	
Men	20 (51.3)	17 (63.0)	3 (25.0)	0.245
BMI (kg/m^2^)	23.6 (13.9–35.5)	22.8 (13.9–35.5)	25.47 (22.5–29.8)	0.181
Comorbidities (ASA PS classification)				
1	2 (5.1)	2 (7.4)	0	
2	17 (43.6)	10 (37.0)	7 (58.3)	
3	20 (51.3)	15 (55.6)	5 (41.7)	0.920
Flap type				
Vertical rectus abdominis (VRAM)	4 (10.2)	4 (14.8)	–	–
Inferior gluteal artery (IGAM)	8 (20.5)	8 (29.6)	–	–
Gracilis	15 (38.4)	15 (55.6)	–	–
VY advancement	3 (7.7)	3 (11.1)	–	–
Approach				
Open	32 (82.1)	25 (92.3)	7 (58.3)	
Laparoscopic	5 (12.8)	2 (7.4)	3 (25.0)	
Robotic	2 (5.1)	0	2 (16.7)	0.304
Neo-adjuvant therapy				
None	5 (12.8)	4 (14.8)	1 (8.3)	
Short course chemo-radiotherapy	3 (7.7)	2 (7.4)	1 (8.3)	
Long-course chemo-radiotherapy	31 (79.5)	21 (77.8)	10 (83.3)	0.318
Recurrence vs primary				
Recurrence	9 (23.1)	6 (22.2)	3 (25.0)	
Primary	30 (76.9)	21 (77.8)	9 (75.0)	0.606

Demographic characteristics all exhibited normal distribution (excluding ‘open/laparoscopic/robotic’). *P* values highlight no difference in cohort demographics.

The predominant patient population for both ELAPE and APR surgeries was classified as ASA 3 (*n* = 15, 55.6% and *n* = 5, 41.7%, respectively). Pre-operative histological analysis indicated that the majority of lesions were malignant, with adenocarcinoma and squamous cell carcinoma being the most common diagnoses.

The vast majority of patients (*n* = 34, 87.2%) received neoadjuvant therapy prior to surgery. Only a small minority (*n* = 5, 12.8%) did not undergo neoadjuvant therapy due to factors such as urgent surgical need, previous radiotherapy, or poor overall health. The majority of operative interventions were performed for primary rectal cancer as opposed to recurrent malignancy (*n* = 30, 76.9% and *n* = 9, 23.1%).

### 3.2 General operative details

**Figure 2:**
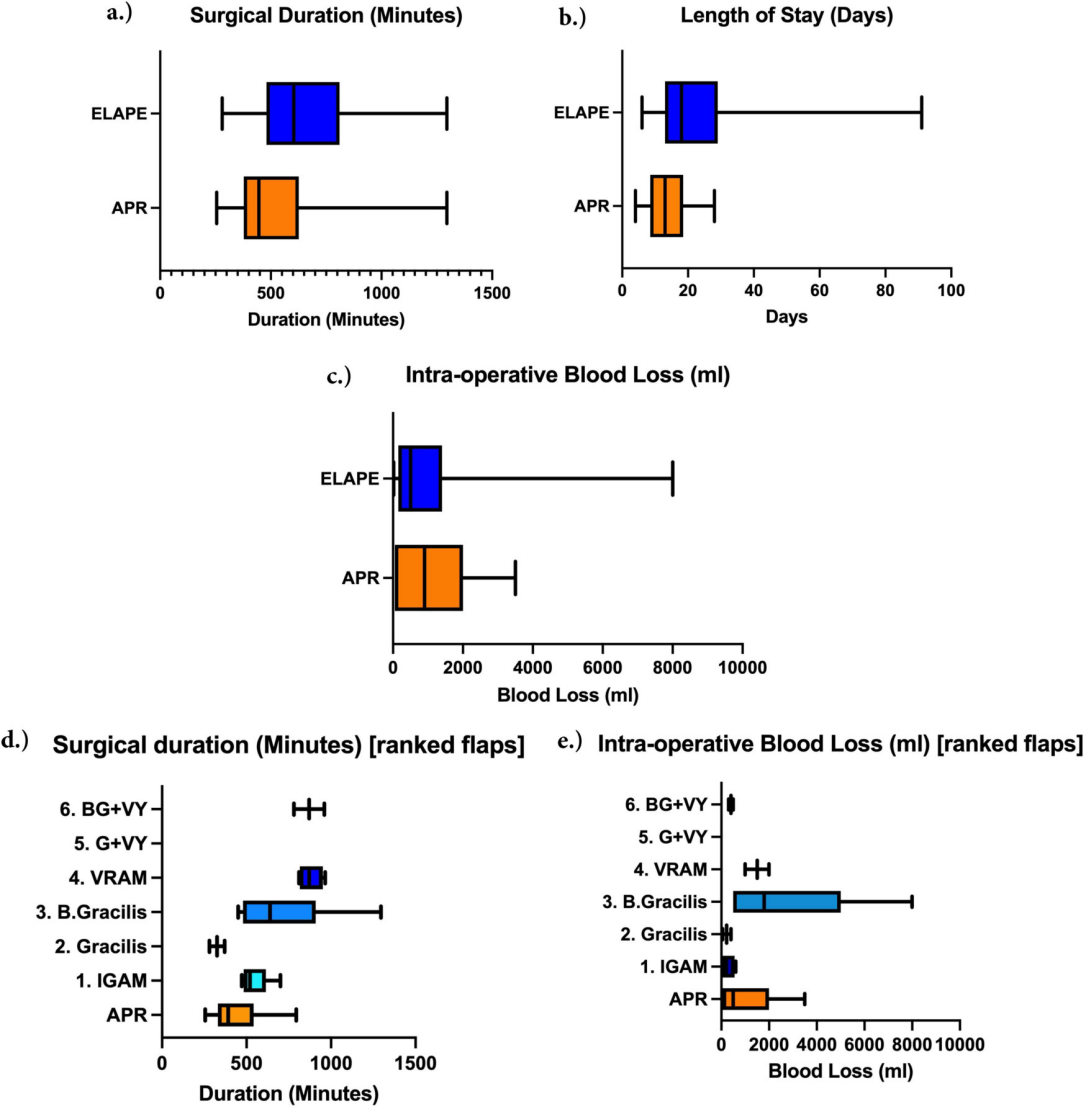
Box-whisker plots of **(a)** the surgical duration (minutes), **(b)** the length of stay (LOS) (days), **(c)** the intra-operative estimated blood loss (ml) for ELAPE and APR cohorts, **(d)** surgical duration (minutes) by flap type grouping, **(e)** intra-operative blood loss (ml) by flap type grouping.

The majority of procedures were performed via an open approach (*n* = 32, 82.1%). Seven cases were conducted using a minimally invasive approach, including two robotic procedures. As our center gains experience with robotic surgery, we are increasingly undertaking more complex cases, such as exenterative procedures. Figure 2 graphically illustrates that the median length of stay (LOS) for APR patients was significantly shorter (13 days) compared to ELAPE patients (18 days), [*P* = 0.037, 95% CI (2.06, 19.49)]. The mean surgical duration for the ELAPE cohort was 648.3 min, and 544.2 min for the APR cohort (Figure 2). No statistically significant difference in surgical duration was observed between the two groups [*P* = 0.301 95% CI (−97.02, 306.00)]. While the mean estimated blood loss (EBL) was higher for the APR cohort comprising 900 ml compared to the ELAPE cohort 500 ml (Figure 2), this difference was not statistically significant [*P* = 0.719 95% CI (−1580, 1301)].

### 3.3 Plastics reconstruction operative details

Table 2 represents flaps used to cover the perineal deffect post exentarative surgery.

**Table 2:** Complications and oncological demographics grouped by flap type.

Flap type	Vertical rectus abdominis myocutaneous (VRAM)	Inferior gluteal artery musculo-cutaneous (IGAM)	Gracilis		VY Advancement
			Unilateral	Bilateral	
Total	4	8	5	10	3
Length of hospital stay post-surgery (days)	28.8 (14–53)	17 (6–40)	19 (13–29)	34.7 (14–91)	23.7 (20–30)
Bilateral	0	0	–	10	2
Unilateral	4	8	5	–	1
Surgical duration (minutes)	879.8 (810–965)	548.8 (472–700)	465 (280–370)	754.1 (450–1,295)	870 (780–960)
Blood loss (ml)	1,500 (1,000–2,000)	274 (20–600)	225 (50–400)	1,942 (300–8,000)	400 (300–500)
Complications: Clavien-Dindo classification					
II	0	3 (37.5)	3 (60.0)	4 (40.0)	–
IIIa	0	3 (37.5)	0	2 (20.0)	–
IIIb	1 (25.0)	0	1 (20.0)	1 (10.0)	–
IVa	1 (25.0)	0	0	0	–
IVb	0	0	0	0	–
Deep surgical site infection					
Conservative management	0	4 (50.0)	0	0	–
Requiring IR drainage	2 (50.0)	2 (25.0)	0	0	–
Flap specific complications					
Superficial infection	1 (25.0)	2 (25.0)	0	1 (10.0)	–
Superficial dehiscence	0	2 (25.0)	0	0	–
Dehiscence requiring theatre/VAC	1 (25.0)	0	0	1 (10.0)	–
Flap necrosis	0	0	0	1 (10.0)	–

Numbers in brackets denote the percentage of the cohort, numbers outside the brackets denote the absolute number within said cohort. The first row's bracketed numbers represent the range of results.

Flaps used in ELAPE perineal reconstruction were as follows: vertical rectus abdominis myocutaneous (VRAM) (*n* = 4, 14.8%), inferior gluteal artery musculocutaneous (IGAM) (*n* = 8, 29.6%), Gracilis flaps (*n* = 15, 55.6%), and V-Y advancement (*n* = 3, 11.1%). Gracilis flaps were used in either a bilateral or unilateral fashion. V-Y flaps were never used independently within the cohort, but instead in conjunction gracilis flaps.

Figure 2 illustrates the total intraoperative blood loss for patients undergoing exenterative surgery with flap reconstruction of perineal defect, categorized by flap complexity (1-6), compared to patients undergoing APR with mesh reconstruction.

When ranked and grouped, against APRs with mesh closure of perineal defects, via analysis of variance, there was found to be a statistically significant difference between the average surgical duration of flap groups against the APR group (*P* = 0.003); The relative confidence intervals for each group were as follows: APR 95% CI: 316.06–566.83, IGAM 95% CI: 481.15–616.35, Bilateral Gracilis 95% CI: 391.75–1039.25, VRAM 95% CI: 766.13–993.37. BG + VY group (95% CI: −273.56 to 2013.56) possessed a wide CI due to *n* = 2 for this flap. This analysis did, however, exclude both the ‘Gracilis + V-Y advancement’ (G + VY) group and ‘Gracilis’ group. The same was not true for EBL, where there was no significant difference between groups (*P* = 0.182) (APR 95% CI: 188.75–1921.25, IGAM 95% CI: 53.06–601.06, Bilateral Gracilis 95% CI: −1315.70 to 6435.70, VRAM 95% CI: −4853.10 to 7853.10, BG + VY 95% CI: −870.62 to 1670.62).

### 3.4 30-day complication rates and flap specific rates

General postoperative complications are displayed in Table 3.

**Table 3:** General complications and oncological demographics of ELAPE/APR cohorts.

Characteristic	Total	ELAPE (with flap)	APR (with mesh recon
Length of hospital stay post-surgery (days)	23.57 (6–91)	24.7 (6–91)	11.9 (4–26)
Complications reported via Clavien–Dindo classification			
II	15 (38.4)	10 (37.0)	5 (41.7)
III	8 (20.5)	8 (29.6)	0
IV	1 (2.6)	1 (3.7)	0
Urinary leak	4 (10.2)	3 (11.1)	1 (8.3)
Small bowel injury	1 (2.6)	1 (3.7)	0
Deep surgical site infection			
Requiring IR drainage	4 (10.2)	4 (14.8)	0
Conservative management	1 (2.6)	1 (3.7)	0
Flap complications			
Superficial infection	4 (10.2)	4 (14.8)	–
Superficial dehiscence	2 (5.1)	2 (7.4)	–
Dehiscence requiring theatre/VAC management	2 (5.1)	2 (7.4)	–
Flap necrosis	1 (2.6)	1 (3.7)	
Negative CRM (R0 resection)	34 (87.2)	24 (88.9)	10 (83.3)
Plastics involvement			
Yes	27 (69.2)	27 (100)	0
No	12 (30.8)	0	12 (100)
Mesh involvement			
Yes	12 (30.8)	0	12 (100)
No	27 (69.2)	27 (100)	0

Numbers in brackets denote the percentage of the cohort, numbers outside the brackets denote the absolute number within said cohort. The first row's bracketed numbers represent the range of results.

Major complications [Clavien-Dindo (CD) III and above] occurred in 9 patients (23.1%), all of whom were in the ELAPE cohort (33.3%). The difference is considered statistically significant (*P* = 0.02). This is expected as ELAPE procedures are multivisceral, involving two or more surgical teams, with reconstruction. No APR cohort patient experienced major complications. Deep surgical site infections requiring interventional radiology (IR) drainage occurred in 14.8% of ELAPE patients (*n* = 4). 11.1% of ELAPE patients (*n* = 3) experienced a urinary leak, compared to 8.3% (*n* = 1) within the APR cohort. Only 1 patient (3.7%) within the ELAPE cohort experienced an iatrogenic small bowel injury. Colorectal resection margin (CRM) positivity was present in 11.1% (*n* = 3) of ELAPE patients and 16.7% (*n* = 2) APR patients.

Complications by flap type are displayed in Table 2. These represent the ELAPE cohort only.

With regards to specific flap complications: 4 (14.8%) were reported as superficial infections; all 4 requiring intravenous (IV) antibiotics (CD II). 2 (7.4%) were reported as superficial dehiscence and underwent conservative management and IV antibiotics (CD II). 2 (7.4%) were reported as dehiscence that required vacuum dressings applied in theatre (CD IIIb). 1 (3.7%) was reported as necrosis, albeit superficial, requiring debridement and vacuum dressing (CD IIIb).

### 3.5 Correlative analysis

Correlative analysis via Spearman's rank correlation co-efficient showed numerous interconnections of variables. Flap Type (ranked by objective complexity) vs. LOS showed strong positive correlation of 0.638 (*P* = 0.003). Flap type (ranked by objective complexity) vs. surgical duration showed moderate positive correlation of 0.539 (*P* = 0.014). Surgical Duration vs. EBL showed strong positive correlation of 0.671 (*P* = 0.0003). Surgical Duration vs. LOS showed strong positive correlation of 0.651 (*P* = 0.002). Clavien-Dindo classification of complications vs. LOS showed moderate positive correlation of 0.530 (*P* = 0.016).

There was little to no correlation between flap type and EBL (0.132, *P* = 0.580) or flap type and Clavien-Dindo complication classification (0.371, *P* = 0.107).

BMI showed negligible and statistically insignificant correlations with LOS (−0.149, *P* = 0.531), Clavien–Dindo complication grade (−0.144 *P* = 0.544), EBL (0.132 *P* = 0.580) and surgical duration (−0.114, *P* = 0.631). Age showed a similar correlative pattern (−0.195, *P* = 0.409; −0.134, *P* = 0.575; −0.130, *P* = 0.586; −0.132, *P* = 0.180 respectively). ASA showed a weak positive correlation with LOS; however this was not statistically significant (0.259, *P* = 0.270), with no meaningful correlations shown against EBL (0.075, *P* = 0.752), Clavien–Dindo (0.068, *P* = 0.775) or surgical duration (0.154, *P* = 0.157).

## 4 Discussion

Locally advanced and recurrent rectal cancer remains a pertinent oncological challenge. Such malignancies contain complexities that require the input of a specialist colorectal cancer MDT services, with a range of multiple specialties contributing to patient care (14). Within these specialties, plastic reconstructive surgery plays a crucial role in enhancing outcomes for patients undergoing extralevator resections (11). Consequently, increased implementation and rigorous study of these reconstructive techniques are essential to assess and refine surgical efficacy, optimise patient recovery, and establish best practice standards.

This retrospective cohort study demonstrates that a developing centre, when equipped with adequate specialist support and necessary expertise, can perform complex perineal reconstructive surgeries safely, achieving patient outcomes comparable to those of higher-volume centres.

### 4.1 Comparison with existing literature

Our outcomes for perineal reconstruction following ELAPE are comparable to those reported by established tertiary and high-volume centers, despite being a developing service. Variability in reporting criteria (e.g., for EBL, LOS, and complication nomenclature) makes direct comparison challenging (4).

For conciseness, we summarize the key outcome metrics from relevant studies alongside our own (Table 4).

**Table 4:** Comparisons of outcomes in our current study with recent published literature.

Study (Year)	Center type	Reconstructions (*n*)	Superficial dehiscence rate	Large dehiscence/intervention rate	Complete flap failure rate	Median LOS (days)
Our study	Large Public	41	7.4%	7.4%	0%	18
Pai et al. (2022) (20)	Tertiary (UK)	27	18.5%	7.4%	N/A	N/A
Jenkins et al. (2023) (12)	Tertiary	N/A	4.0%	N/A	N/A	N/A
Barker et al. (2024) (21)	Large Public	N/A	9.1%	N/A	3.6%	11

Our superficial dehiscence rate (7.4%) is lower than the minor dehiscence rate reported by Pai et al. (18.5%) (20) and comparable to Barker et al. (9.1%) (21). The rate of larger dehiscence requiring intervention (7.4%) aligns with the 7.4% rate reported by Pai et al. (20). Importantly, no patients in our cohort experienced complete flap failure, which contrasts favourably with the 3.6% reported by Barker et al. (21). Studies from high-volume centers in Dresden (8) and Maastricht (22) report overall flap site complication rates ranging from 21.3% to 36.1% and perineal wound dehiscence rates of 26.8%, respectively. Although some larger centers report faster median ELAPE operative times (e.g., 486 min vs. our 604 min) (8), our complication profile remains favourable.

In order to accurately stratify complications and correlate these to flap type, We employed a ranking system, based on expert opinion and established literature (23–30), to stratify flaps by relative operative complexity. This supported the observed associations between flap type, operative parameters (like operative duration), and postoperative outcomes. However, caution should be exercised in interpreting these results; correlation does not equate to causation, and the observed associations do not imply a direct causal link between flap type and length of stay or complication rates.

### 4.2 Limitations

We acknowledge the limitations of this study. Our experience of reconstructive perineal surgery in the context of oncological resections is rapidly increasing, therefore more advanced multivariate analysis is not yet feasible, excluding simple correlative analyses.

Multivariate regression analysis was explored to further evaluate factors contributing to differences in postoperative length of stay. However, given the limited number of patients available for modelling and the high proportion of individuals who received neoadjuvant chemoradiotherapy (87.2% of the cohort), the resulting subgroup sizes were insufficient to support stable or reliable multivariable estimates. Attempts to construct linear and Cox regression models resulted in non-estimable coefficients and infinite ratio statistics, indicating over-stratification relative to sample size rather than true underlying effects. Similarly, stratified comparisons according to neoadjuvant therapy status were considered; however, subdivision of our patient cohort into treatment strata produced groups too small to allow meaningful statistical interpretation. As such, the analyses presented focus on unstratified comparisons, which remain valid for describing observable trends within the available data.

While recognising that correlation does not imply causation, Spearman's rank correlation was employed to explore potential associations between perioperative variables and postoperative length of stay in the context of a cohort not large enough to support robust multivariable modelling. In lieu of formal multivariate regression, this approach enabled the identification of patterns suggestive of contributing or confounding factors, providing a clearer understanding of how surgical complexity, operative duration, and postoperative complications may relate to recovery trajectories within this dataset.

The use of the greater omentum flap was not consistently documented in the operative records, and therefore this variable was absent for the entire cohort. Typically these were performed in extensive exenteration surgery to pack the pelvis and prevent empty pelvis syndrome. As this represents a systematic absence of data rather than sporadic missingness within an otherwise complete dataset, standard methods for handling missing data (such as imputation or sensitivity analyses) were not applicable. Instead, this parameter was excluded from comparative modelling, and all analyses were conducted on the fully observed variables. This limitation reflects the underlying documentation practices during the study period rather than an omission of the research protocol.

We also acknowledge the inherent limitations of a retrospective cohort study including, but not limited to, possible selection bias and limited generalisability. Because of this, we understand that more data will be required to assess the success of perineal reconstructive surgery following rectal cancer surgery.

### 4.3 Clinical implications and relevance to multidisciplinary practice

Access to the complex colorectal cancer MDT and plastic surgery team was critical to the process of patient-tailored flap planning at our center. Co-ordination with other centers enabled joint plastics-colorectal clinics for patient examination and flap evaluation. While we recognize that such advanced, resource-intensive services may not be present at all large public hospitals, our study demonstrates the potential for developing services to perform these complex operations safely.

The successful implementation of these procedures in a large public hospital context, with outcomes comparable to tertiary centers, underscores the value of robust MDT collaboration. This includes not only the surgical specialties but also oncology and nursing services, which are vital for managing the complex post-operative course. The ability to safely offer these complex reconstructions outside of established, high-volume tertiary centers is crucial for addressing capacity issues and potentially reducing waiting times in the broader healthcare system.

### 4.4 Clear clinical takeaway

This study provides evidence that complex flap reconstructions following extralevator abdominoperineal excision for locally advanced rectal cancer can be performed safely and effectively in large public hospitals, provided there is sufficient multidisciplinary collaboration and specialized expertise in place.

## 5 Conclusion

This retrospective cohort study, conducted at a large public hospital, demonstrates the safety and feasibility of complex perineal flap reconstruction following Extralevator Abdominoperineal Excision (ELAPE) for locally advanced rectal cancer in a non-tertiary setting. The observed flap complication rates are comparable to those reported by higher-volume centers, indicating favorable patient outcomes.

The success of these complex procedures critically relies on multidisciplinary cooperation, specifically the integration and resource provision of a complex colorectal MDT and specialist plastic reconstructive surgery team, supported by rigorous preoperative planning.

To solidify these findings and establish standardized protocols, there is a clear necessity for prospective, multicenter validation across various healthcare settings. This future research will better define the optimal delivery and resource allocation required to maintain high-quality care for this challenging patient cohort.

## Data Availability

The original contributions presented in the study are included in the article/Supplementary Material, further inquiries can be directed to the corresponding author/s.
